# Endoscopic Retrograde Cholangiopancreatography Treatment of Cholecystitis: Possible? Yes; Practical??

**DOI:** 10.1155/DTE.1.51

**Published:** 1994

**Authors:** Jerome H. Siegel, Franklin E. Kasmin, Seth A. Cohen

**Affiliations:** 1 Beth Israel Medical Center North Division New York NY USA; 2 Attending Medicine-Gastroenterology Beth Israel Medical Center New York NY USA; 3 F.A.C.P. F.A.C.G. 60 East End Avenue New York NY 10028 USA

## Abstract

Classically, until now, the management of cholecystitis has consisted of immediate and judicious clinical assessment of the affected patient, interpolating into the assessment of the physical findings and results from appropriate laboratory, x-ray, and scanning techniques (sonography and scintigraphy) to formulate a clinical impression. Usually, after the diagnosis has been established, the patient is subjected to a cholecystectomy, although the timing of the surgery may vary depending on the clinical condition of the patient. Alternatives to this management (cholecystectomy, medical management) scheme have been suggested, but these are dependent upon the clinical condition ofthe patient and considerations of risks. Percutaneous drainage of the gallbladder or cholecystostomy is sufficient enough to provide drainage, relieve obstruction, and the consequences of infection, *i.e.*, sepsis, and prevent perforation. A contributory role of endoscopic retrograde cholangiopancreatography (ERCP) in this schema has not been a consideration. An ERCP is rarely employed for therapy (or  diagnosis) when cholecystitis is suspected but it might assume a more significant role if it is considered an efficacious alternative in specific conditions. We have had the unusual experience of managing 11 patients with cholecystitis employing ERCP and its therapeutic modalities, i.e., sphincterotomy, selective cannulation of the cystic duct, and relieving obstruction of that structure by catheter displacement of an obstructing stone. Endoscopic techniques providing decompression of the gallbladder are described, and the feasibility of utilizing endoscopic procedures for treatment of cholecystitis will be given consideration.


*Diagnostic and Therapeutic Endoscopy*, Vol. 1, pp. 51-56
Reprints available directly from the publisher
Photocopying permitted by license only

© 1994 Harwood Academic Publishers GmbH
Printed in Malaysia

### Endoscopic Retrograde Cholangiopancreatography Treatment of Cholecystitis: Possible? Yes; Practical??

JEROME H. SIEGEL*, FRANKLIN E. KASMIN** and SETH A. COHEN**

**Beth Israel Medical Center North Division, New York, NY*
***Attending, Medicine-Gastroenterology, Beth Israel Medical Center, New York, NY*

*(Received August 4, 1993; in final form December 6, 1993)*

Classically, until now, the management of cholecystitis has consisted of immediate and judicious clinical assessment of the affected patient, interpolating into the assessment of the physical findings and results from appropriate laboratory, x-ray, and scanning techniques (sonography and scintigraphy) to formulate a clinical impression. Usually, after the diagnosis has been established, the patient is subjected to a cholecystectomy, although the timing of the surgery may vary depending on the clinical condition of the patient. Alternatives to this management (cholecystectomy, medical management) scheme have been suggested, but these are dependent upon the clinical condition of the patient and considerations of risks. Percutaneous drainage of the gallbladder or cholecystostomy is sufficient enough to provide drainage, relieve obstruction, and the consequences of infection, *i.e.*, sepsis, and prevent perforation. A contributory role of endoscopic retrograde cholangiopancreatography (ERCP) in this schema has not been a consideration. An ERCP is rarely employed for therapy (or diagnosis) when cholecystitis is suspected but it might assume a more significant role if it is considered an efficacious alternative in specific conditions. We have had the unusual experience of managing 11 patients with cholecystitis employing ERCP and its therapeutic modalities, i.e., sphincterotomy, selective cannulation of the cystic duct, and relieving obstruction of that structure by catheter displacement of an obstructing stone. Endoscopic techniques providing decompression of the gallbladder are described, and the feasibility of utilizing endoscopic procedures for treatment of cholecystitis will be given consideration.

**KEY WORDS:** cholecystitis, cholecystectomy, ERCP (endoscopic retrograde cholangiopancreatography), sphincterotomy, stents (prostheses).

### INTRODUCTION

Use of the time-honored surgical approach to cholecystitis, cholecystectomy, has not been challenged, ostensibly because the results of simple extirpation of the gallbladder in nonemergency or elective situations have been acceptable (Sallej and Balasegarem, 1974). Extenuating circumstances, i.e., cardiovascular or pulmonary instability, may preclude open cholecystectomy or even laparoscopic cholecystectomy. In compromised patients considered at high risk, percutaneous drainage of the gallbladder or surgical cholecystostomy are accepted prudent alternatives employed in the management of acute cholecystitis when surgery is contraindicated (Pearse *et al.*, 1984).

Since the introduction of endoscopic retrograde cholangiopancreatography (ERCP) with sphincterotomy, and removal of common bile duct stones, endoscopic access to the cystic duct has become a reality. Consequently, access into the gallbladder has provided therapeutic alternatives for the management of cholecystitis (Kasmin *et al.*, 1993; Tamada *et al.*, 1991; Stabenow-Lonbauer *et al.*, 1991). These accomplishments became a reality due to the introduction of special hydrophilic guide wires, especially the Glidewire[R] produced by Terumo and distributed by Microvasive Inc., and the Tracer wire[R] manufactured by Wilson-Cook, Inc. Although endoscopic entry into the gallbladder is infrequent, this accomplishment has prompted endoscopists in certain reports, to infer that endoscopic techniques might be considered for the treatment of patients with cholelithiasis and cholecystitis (Tamada *et al.*, 1991). When new technology is considered for general use, it must stand up to the accepted standard of care, providing safe, effective, and comparable results, while, at the same time offering reduced morbidity, mortality, and hos-

Address for correspondence: Jerome H. Siegel, M.D., F.A.C.P., F.A.C.G., 60 East End Avenue, New York, NY 10028.

pitalization. The newer endoscopic techniques appear to satisfy the above criteria in certain situations.

Although cannulation of the cystic duct is often fortuitous, deliberate, selective cannulation of that structure is possible in some patients and might be a consideration as an alternative treatment for patients who are at great surgical risk. In this report, we describe the techniques we have employed for selective cannulation of the cystic duct and gallbladder, manipulation of cystic duct stones, and the insertion of a prosthesis into the gallbladder. Management strategies for a clinical condition formerly treated by surgery but amenable to endoscopic therapy, in selected cases, will be discussed.

### METHODS

After completing an ERCP and selectively cannulating the common bile duct, the cystic duct junction and/or its insertion is identified. The cannula, which is preferentially radiopaque, is advanced into the distal end of the bile duct, and an attempt is made to enter the cystic duct orifice. This maneuver is best appreciated on the fluoroscope when using a radiopaque cannula. While manipulating the catheter, contrast material is injected into the bile duct in an attempt to identify selective filling of the cystic duct. Should an obstructing stone be present in that orifice or in the cystic duct, it can be displaced by forceful injection of contrast material. Filling of the gallbladder rules out obstruction to the cystic duct. If gallstones are appreciated in the gallbladder in the clinical setting of cholecystitis, drainage of the gallbladder may be possible by one of four endoscopic methods: (1) Sphincterotomy, which can provide free drainage of the cystic duct and gallbladder; (2) injection of contrast into the cystic duct to dislodge an obstructing stone; (3) manipulation of the cystic duct using a catheter-guide wire assembly to dislodge the stone; and, (4) placement of a prosthesis into the gallbladder (Fig. 1).

The technique for performing endoscopic sphincterotomy (ES) has been previously described and will not be discussed here. ES was performed in all patients to facilitate repeated entries to the bile duct and to avoid manipulation of the pancreas. Selective cannulation of the cystic duct and insertion of a guidewire is possible using a catheter which will accept a 0.035 inch guidewire. With a cannula or catheter in place in the cystic duct orifice or within the cystic duct itself, a guidewire can be inserted through that accessory, facilitating entry through the valves of Heister into the neck and body of the gallbladder. If a guidewire can be placed into the gallbladder, a balloon catheter or basket can be used to extract stones from the gallbladder or cystic duct. The technique for placing a prosthesis into the gallbladder is similar to that of placing a prosthesis into the bile duct or pancreatic duct with one exception. That exception is the preferred pigtail configuration of a prosthesis we employ for insertion into the gallbladder. It is the authors' preference to use a double pigtail prosthesis for gallbladder decompression rather than a straight prosthesis (preferred by the authors for malignant strictures).

### PATIENTS

Seventeen patients, 12 females, ages 43–90 years, mean 72.4 years, were referred for the evaluation or treatment of cholecystitis. All patients presented with upper right quadrant pain, tenderness to palpation, and fever (C 37.5–38). An elevated serum white blood count was present in 15 (one patient with acalculous cholecystitis undergoing chronic parenteral nutrition and one patient with diabetes did not have an increased count). Radioscintigraphic hepatobiliary scans were positive in all patients, with failure to fill the gallbladder in 12 patients and failure to empty the gallbladder in 5. Ultrasonography or computed tomography demonstrated cholelithiasis in 15 patients. Cultures were obtained in 3 patients and these cultures grew *E. Coli.* Parenteral antibiotics were administered to all patients, 14 had antibiotics before ERCP, and all received antibiotics after the procedure.

### RESULTS

Altogether, endoscopic techniques were selected as the only invasive method of treating cholecystitis in 11 affected patients (Table 1). Selective cannulation of the cystic duct was attempted in 17 patients, for a success rate of 65%. Prostheses were inserted into the gallbladders of 4 patients in whom this was attempted following sphincterotomy (Fig. 3). Four other patients were treated with sphincterotomy alone (no evidence of cystic duct stone, no prosthesis attempted), and three patients were treated with the combination of sphincterotomy and selective cannulation of the cystic duct and injection of contrast under pressure into that structure. In 3 other patients, injection was attempted, but a stone could not be dislodged. No patients required surgery and no complications occurred with follow-up of 10 months to 8 years. Stents were changed at 6 months in 2 patients, and the 2 other patients with stents died of liver disease at 14 and 24 months, respectively without evidence of recurrent cholecystitis. No cholecystitis or cholangitis occurred in our long term fol-

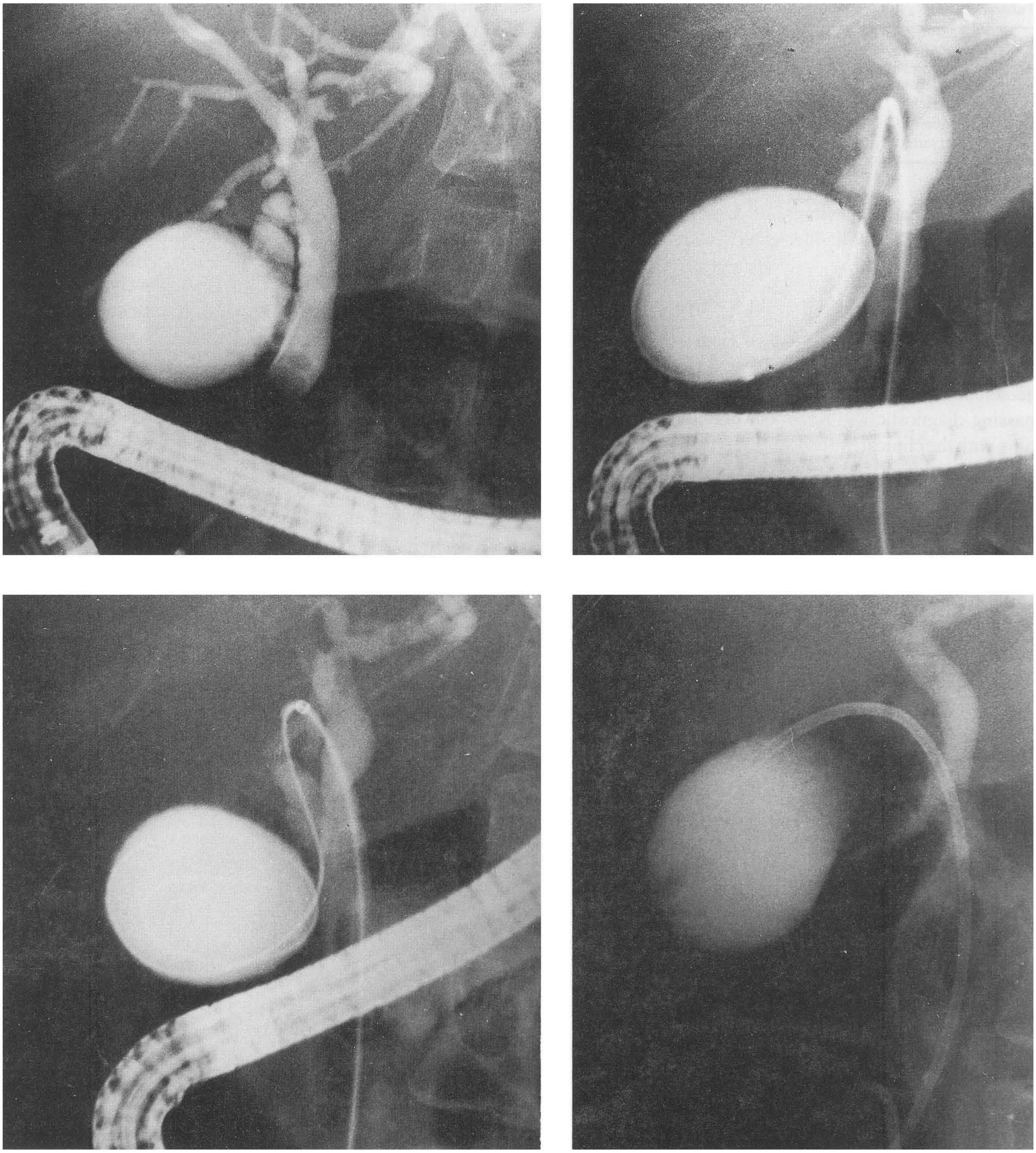

**Figure 1** Upper left, Cholangiogram obtained from a patient with acalculous cholecystitis secondary to chronic parenteral nutrition. Upper right, Glidewire[R] advanced into the cystic duct and gallbladder. Lower left, Dilating catheter advanced over guide wire into the cystic duct. Lower right, Double pigtail stent in the gallbladder.

**Table 1** Cholecystitis—Endoscopic Treatment

| *Procedure* | *No. of Patients* |
|---|---|
| Sphincterotomy | 11 |
| Prostheses | 4 |
| Injection of Contrast | 3 |
| Sphincterotomy only | 4 |

low up of the patients with gallbladder prostheses despite the presence of a gallbladder-enteric communication.

### DISCUSSION

The accepted treatment of acute and recurrent acute/chronic cholecystitis should include an all encompassing diagnostic and treatment plan, obviously beginning with the clinical evaluation of the patient. Appropriate laboratory, radiographic, and scanning studies are obtained early in the patient's clinical course to confirm the admitting diagnosis or impression. If the patient's condition responds to conservative medical management consisting of appropriate antibiotics and supportive therapy, cholecystectomy, the accepted standard of care, can be scheduled as an elective procedure. Most surgeons prefer operating after a brief interval, allowing for an appropriate response to therapy, i.e., resolution of the inflammatory process and reduction of edema, although emergency cholecystectomy is not an uncommon operative procedure (Jarvinen and Hastbacka, 1990, Van der Linden and Edlund, 1981).

Patients considered at greater surgical risk may be managed differently. A more pragmatic approach is employed in patients at risk, and more time is devoted to clinical observation and assessment of the treatment plan while contemplating surgical intervention. Subsequent removal of the gallbladder by cholecystectomy, either open or laparoscopic, is carried out, or, alternatively, appropriate drainage can be provided by radiologic or surgical cholecystostomy (Glenn, 1977).

When incorporating the above clinical schema into practice, most patients usually survive the potential serious complications attendant with cholecystitis and surgical intervention. Consideration of alternative therapeutic maneuvers is not paramount when established modalities have been proven effective. However, in some situations endoscopists are called upon to provide a diagnostic cholangiogram rendered through ERCP to determine the patency of the cystic duct when a HIDA scan is equivocal or consistent with an obstruction. If selective cannulation and manipulation of the cystic duct is accomplished

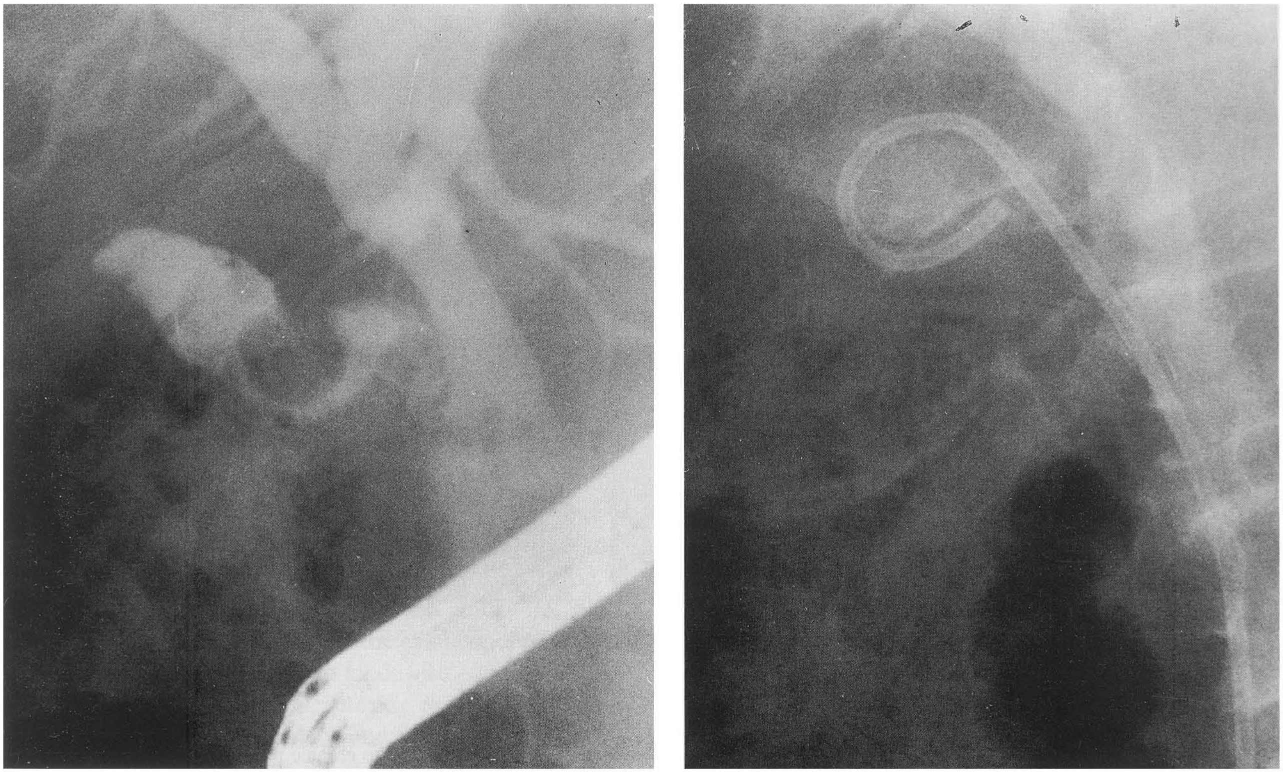

**Figure 2** Left, Stone seen in the neck of the gallbladder. Right, Pigtail prosthesis in gallbladder around stone.

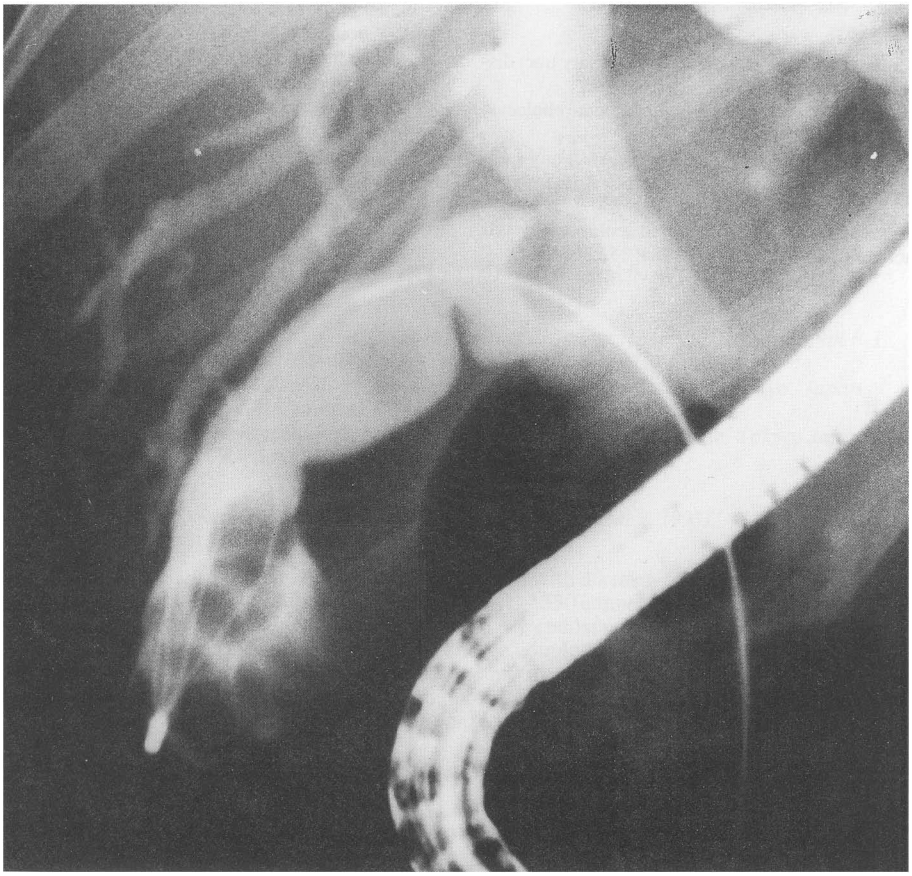

**Figure 3** Basket seen in gallbladder entrapping stones for removal.

during the endoscopic procedure, obstructing stones are usually dislodged by injecting contrast directly against the offending stone. Having accessed the cystic duct and gallbladder, innovative endoscopists have left drains in the gallbladder to adequately decompress and drain this infected organ with beneficial effects (Kasmin *et al.*, 1993; Tamada *et al.* 1991). Nasobiliary drains have been left in the gallbladder to perfuse agents to dissolve stones. (Stabenow-Lonbauer *et al.*, 1991; Soehendra *et al.*, 1990). One complication has been reported using a standard guidewire to access the gallbladder, but none have occurred using the new hydrophilic wires (Soehendra *et al.*, 1990).

The notion that a cholecysto-enteric communication might perpetuate an inflammatory process or provoke infection has not been confirmed by us or by other authors, nor has this notion been borne out in the literature. In the authors' experience, which includes extended observation of our patients, the presence of a foreign body or prothesis in the gallbladder in communication with the duodenum has not contributed to worsening of the cholecystitis, or to perforation, hydrops, or gangrenous changes. In fact, none of our patients required early intervention. Only one patient underwent elective cholecystectomy, while the others have been followed by their referring physicians. Two patients have undergone stent exchanges at 6 months, while two other patients with gallbladder stents died at 14 and 24 months of unrelated causes.

Although endoscopic access to the cystic duct and gallbladder is possible, such a maneuver must still be considered an experimental form of therapy. The number of reported cases of cholecystitis treated endoscopically is small, and the follow-up is variable. Additionally, the skill of endoscopists is not the same, and this therapy may not be a practical alternative in centers where endoscopic expertise is limited. However, if the procedure is safe and offers results comparable to those of accepted techniques in certain situations with reduced morbidity, it warrants consideration as an alternative therapy.

The authors offer this treatment modality for application in limited situations as a safe alternative in an acutely ill, compromised patient. In the foreseeable future, after more data has been accrued and assessed, endoscopy may be included in the management scheme of cholecystitis.

### REFERENCES

Glenn F. Cholecystostomy in the high risk patient with biliary tract disease. *Ann Surg*, 1977;185(2):185–191.

Jarvinen H. J., Hastbacka J.: Early cholecystectomy for acute cholecystitis. *Ann Surg*, 1990;191(4):501–505.

Kasmin E. E., Cohen S. A., Siegel J. H.: Medical cholecystectomy—endoscopic management of cholecystitis. *Gastrointest Endoscopy*, 1993;39(2):A337.

Pearse D. M. *et. al.*: Percutaneous cholecystotomy in acute cholecystitis and common duct obstruction. *Radiology*, 1984; 152:365–367.

Sallej H. B. M., Balasegarem M.: Treatment of acute cholecystitis by routine urgent operation. *Br J Surg*, 1974; 61:705–708.

Soehendra N. *et al.*: ESWL and gallstone dissolution with MTBE via a naso-vesicular catheter. *Endoscopy*, 1990;22(4):176–9.

Stabenow-Lonbauer U. *et. al.* Lysis of gallstones with methyl tert-butyl ether: percutaneous, transhepatic, or transpapillary? Deutsche Med. Wochenschrift, 1991;116(41):1537–1542.

Tamada K. *et. al.* Efficacy of endoscopic retrograde cholecystoendoprosthesis for cholecystitis. *Endoscopy*, 1991;23(1):2–3.

Van Der Linden W., Edlund G.: Early versus delayed cholecystectomy the effect of a change in management. *Br J Surg* 1981;68:753–757.

